# 3-(2-Methyl­phen­yl)-3a,4-dihydro-3*H*-chromeno[4,3-*c*]isoxazole-3a-carbo­nitrile

**DOI:** 10.1107/S1600536812051732

**Published:** 2013-01-04

**Authors:** G. Suresh, J. Srinivasan, M. Bakthadoss, S. Aravindhan

**Affiliations:** a Department of Physics, Presidency College (Autonomous), Chennai 600 005, India; b Department of Organic Chemistry, University of Madras, Chennai 600 025, India

## Abstract

In the title compound, C_18_H_14_N_2_O_2_, the pyran ring of the chromeno ring system has a half-chair conformation, and the dihedral angle between its mean plane and the benzene ring is 5.3 (2)°. The isoxazole ring forms a dihedral angle of 74.6 (2)° with the attached benzene ring and is inclined to the mean plane of the chromeno ring system by 15.06 (19)°. In the crystal, there are no significant inter­molecular inter­actions.

## Related literature

For the biological importance of 4*H*-chromene derivatives, see: Cai (2007 ▶, 2008 ▶); Cai *et al.* (2006 ▶); Gabor (1988 ▶); Brooks (1998 ▶); Valenti *et al.* (1993 ▶); Hyana & Saimoto (1987 ▶); Tang *et al.* (2007 ▶). For related structures, see: Gangadharan *et al.* (2011 ▶); Swaminathan *et al.* (2011 ▶).
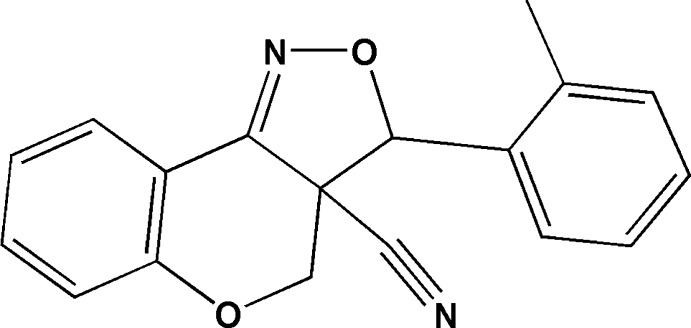


## Experimental

### 

#### Crystal data

C_18_H_14_N_2_O_2_
*M* *_r_* = 290.31
Orthorhombic, 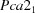

*a* = 19.326 (3) Å
*b* = 10.7866 (17) Å
*c* = 6.9072 (11) Å
*V* = 1439.9 (4) Å^3^
*Z* = 4
Mo *K*α radiation
μ = 0.09 mm^−1^
*T* = 298 K
0.35 × 0.25 × 0.15 mm

#### Data collection

Bruker APEXII CCD area-detector diffractometer
Absorption correction: multi-scan (*SADABS*; Bruker, 2008 ▶) *T*_min_ = 0.970, *T*_max_ = 0.987
4742 measured reflections
1750 independent reflections
1100 reflections with *I* > 2σ(*I*)
*R*_int_ = 0.047

#### Refinement

*R*[*F* ^2^ > 2σ(*F* ^2^)] = 0.059
*wR*(*F* ^2^) = 0.146
*S* = 1.09
1750 reflections
200 parameters
1 restraint
H-atom parameters constrained
Δρ_max_ = 0.21 e Å^−3^
Δρ_min_ = −0.17 e Å^−3^

### 

Data collection: *APEX2* (Bruker, 2008 ▶); cell refinement: *SAINT* (Bruker, 2008 ▶); data reduction: *SAINT*; program(s) used to solve structure: *SHELXS97* (Sheldrick, 2008 ▶); program(s) used to refine structure: *SHELXL97* (Sheldrick, 2008 ▶); molecular graphics: *ORTEP-3* (Farrugia, 2012 ▶); software used to prepare material for publication: *SHELXL97* and *PLATON* (Spek, 2009 ▶).

## Supplementary Material

Crystal structure: contains datablock(s) I, global. DOI: 10.1107/S1600536812051732/su2544sup1.cif

Structure factors: contains datablock(s) I. DOI: 10.1107/S1600536812051732/su2544Isup2.hkl

Supplementary material file. DOI: 10.1107/S1600536812051732/su2544Isup3.cml

Additional supplementary materials:  crystallographic information; 3D view; checkCIF report

## Figures and Tables

**Fig. 1.**
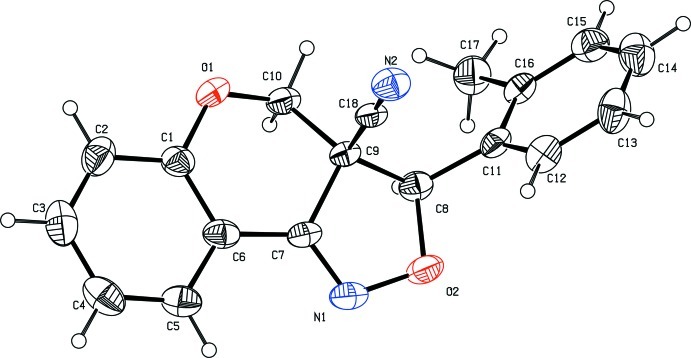
The molecular structure of the title compound, with the atom labeling. Displacement ellipsoids are drawn at the 30% probability level.

## supplementary crystallographic information

### Comment

4*H*-Chromenes are biologically important compounds used as synthetic ligands for drug designing and discovery process. They exhibit numerous biological and pharmacological properties such as anti-viral, anti-fungal, anti-inflammatory, anti- diabetic, cardionthonic, anti anaphylactic and anti-cancer activity (Cai, 2008; Cai, 2007; Cai *et al.*, 2006; Gabor, 1988; Brooks, 1998; Valenti *et al.*, 1993; Hyana & Saimoto, 1987; Tang *et al.*, 2007). We report herein on the synthesis of a new chromeno compound and its crystal structure.

The molecular structure of the title molecule is illustrated in Fig. 1. In the chromeno ring system, the dihedral angle between the mean plane of the pyran ring, which has a half-chair conformation, and the benzene ring is 5.3 (2)°. The dihedral angle between the mean plane of the chromeno ring system and isoxazole ring is 15.06 (19)°. The isoxazole ring also forms a dihedral angle of 74.6 (2)° with the the benzene ring (C11—C16). The geometric parameters of the title molecule agree well with those reported for closely related structures (Gangadharan *et al.*, 2011; Swaminathan *et al.*, 2011).

In the crystal, there are no significant intermolecular interactions.

### Experimental

NCS (4 mmol) was added pinch wise over 3h to a solution of (*E*)-2-((2-((*E*)-(hydroxyimino)methyl)phenoxy)methyl)-3-*o*-tolylacrylonitrile(2 mmol) in CCl_4_ at 273 - 283 K. After Et_3_N (4 mmol) was added to the reaction mixture which was stirred at room temperature for 2 h. After completion of the reaction, the mixture was evaporated under reduced pressure and the resulting crude mass was diluted with water (15 ml) and extracted with ethyl acetate (3 × 15 ml). The combined organic layers washed with brine (2 × 10 ml) and dried over anhydrous Na_2_SO_4_. The organic layer was evaporated and purified by column chromatography (silica gel 60–120 mesh; 7% EtOAc in hexanes) to provide the desired title product as a colourless solid. Crystals suitable for X-ray diffraction were obtained by slow evaporation of a solution of the title compound in ethyl acetate at room temperature.

### Refinement

All the hydrogen atoms were placed in calculated positions and refined as riding atoms: C—H = 0.93–0.98 Å with *U*_iso_(H) = 1.5U_eq_(C) for methyl group and = 1.2U_eq_(C) for other groups. In the final cycles of refinement, in the absence of significant anomalous scattering effects, Friedel pairs were merged and Δf " set to zero.

### Crystal data

**Table:**

C_18_H_14_N_2_O_2_	*F*(000) = 608
*M**_r_* = 290.31	*D*_x_ = 1.339 Mg m^−^^3^
Orthorhombic, *P**c**a*2_1_	Mo *K*α radiation, λ = 0.71073 Å
Hall symbol: P 2c -2ac	Cell parameters from 1750 reflections
*a* = 19.326 (3) Å	θ = 1.9–27.7°
*b* = 10.7866 (17) Å	µ = 0.09 mm^−^^1^
*c* = 6.9072 (11) Å	*T* = 298 K
*V* = 1439.9 (4) Å^3^	Orthorhombic, colourless
*Z* = 4	0.35 × 0.25 × 0.15 mm

### Data collection

**Table:**

Bruker APEXII CCD area-detector diffractometer	1750 independent reflections
Radiation source: fine-focus sealed tube	1100 reflections with *I* > 2σ(*I*)
Graphite monochromator	*R*_int_ = 0.047
ω and φ scans	θ_max_ = 27.7°, θ_min_ = 1.9°
Absorption correction: multi-scan (*SADABS*; Bruker, 2008)	*h* = −24→25
*T*_min_ = 0.970, *T*_max_ = 0.987	*k* = −10→13
4742 measured reflections	*l* = −8→6

### Refinement

**Table:**

Refinement on *F*^2^	Primary atom site location: structure-invariant direct methods
Least-squares matrix: full	Secondary atom site location: difference Fourier map
*R*[*F*^2^ > 2σ(*F*^2^)] = 0.059	Hydrogen site location: inferred from neighbouring sites
*wR*(*F*^2^) = 0.146	H-atom parameters constrained
*S* = 1.09	*w* = 1/[σ^2^(*F*_o_^2^) + (0.0456*P*)^2^ + 0.5663*P*] where *P* = (*F*_o_^2^ + 2*F*_c_^2^)/3
1750 reflections	(Δ/σ)_max_ < 0.001
200 parameters	Δρ_max_ = 0.21 e Å^−^^3^
1 restraint	Δρ_min_ = −0.17 e Å^−^^3^

### Special details

**Table:**

Geometry. All e.s.d.'s (except the e.s.d. in the dihedral angle between two l.s. planes) are estimated using the full covariance matrix. The cell e.s.d.'s are taken into account individually in the estimation of e.s.d.'s in distances, angles and torsion angles; correlations between e.s.d.'s in cell parameters are only used when they are defined by crystal symmetry. An approximate (isotropic) treatment of cell e.s.d.'s is used for estimating e.s.d.'s involving l.s. planes.
Refinement. Refinement of *F*^2^ against ALL reflections. The weighted *R*-factor *wR* and goodness of fit *S* are based on *F*^2^, conventional *R*-factors *R* are based on *F*, with *F* set to zero for negative *F*^2^. The threshold expression of *F*^2^ > σ(*F*^2^) is used only for calculating *R*-factors(gt) *etc*. and is not relevant to the choice of reflections for refinement. *R*-factors based on *F*^2^ are statistically about twice as large as those based on *F*, and *R*- factors based on ALL data will be even larger.

### Fractional atomic coordinates and isotropic or equivalent isotropic displacement parameters (Å^2^)

**Table:**

	*x*	*y*	*z*	*U*_iso_*/*U*_eq_	
C1	0.8559 (2)	−0.2941 (4)	0.6812 (9)	0.0493 (12)	
C2	0.8529 (2)	−0.4218 (5)	0.6834 (12)	0.0659 (16)	
H2	0.8639	−0.4667	0.5727	0.079*	
C3	0.8336 (3)	−0.4824 (5)	0.8491 (13)	0.0725 (18)	
H3	0.8311	−0.5685	0.8502	0.087*	
C4	0.8177 (2)	−0.4155 (6)	1.0168 (11)	0.0717 (18)	
H4	0.8053	−0.4572	1.1294	0.086*	
C5	0.8204 (2)	−0.2907 (5)	1.0155 (9)	0.0597 (14)	
H5	0.8093	−0.2470	1.1274	0.072*	
C6	0.8397 (2)	−0.2254 (5)	0.8478 (8)	0.0494 (13)	
C7	0.84595 (19)	−0.0931 (5)	0.8391 (7)	0.0421 (12)	
C8	0.8522 (2)	0.0964 (4)	0.6809 (8)	0.0457 (11)	
H8	0.8093	0.1013	0.6058	0.055*	
C9	0.88122 (19)	−0.0358 (4)	0.6634 (7)	0.0384 (10)	
C10	0.8602 (2)	−0.1104 (5)	0.4876 (8)	0.0482 (12)	
H10A	0.8109	−0.1007	0.4659	0.058*	
H10B	0.8842	−0.0789	0.3746	0.058*	
C11	0.8981 (2)	0.2017 (4)	0.6220 (7)	0.0434 (12)	
C12	0.9523 (2)	0.2369 (4)	0.7453 (9)	0.0558 (14)	
H12	0.9572	0.1988	0.8653	0.067*	
C13	0.9984 (2)	0.3274 (5)	0.6900 (11)	0.0674 (17)	
H13	1.0337	0.3520	0.7733	0.081*	
C14	0.9919 (3)	0.3817 (5)	0.5095 (13)	0.0744 (19)	
H14	1.0239	0.4406	0.4686	0.089*	
C15	0.9383 (3)	0.3483 (5)	0.3917 (9)	0.0618 (14)	
H15	0.9341	0.3865	0.2716	0.074*	
C16	0.8901 (2)	0.2600 (4)	0.4442 (8)	0.0474 (12)	
C17	0.8323 (3)	0.2283 (5)	0.3092 (9)	0.0668 (15)	
H17A	0.7888	0.2425	0.3726	0.100*	
H17B	0.8357	0.1426	0.2726	0.100*	
H17C	0.8353	0.2793	0.1957	0.100*	
C18	0.9575 (2)	−0.0430 (4)	0.6899 (7)	0.0412 (10)	
N1	0.82063 (19)	−0.0141 (4)	0.9557 (7)	0.0556 (11)	
N2	1.01564 (18)	−0.0484 (4)	0.7066 (7)	0.0570 (11)	
O1	0.87563 (16)	−0.2367 (3)	0.5112 (6)	0.0556 (9)	
O2	0.83494 (17)	0.1062 (3)	0.8863 (5)	0.0582 (9)	

### Atomic displacement parameters (Å^2^)

**Table:**

	*U*^11^	*U*^22^	*U*^33^	*U*^12^	*U*^13^	*U*^23^
C1	0.038 (2)	0.057 (3)	0.054 (3)	−0.001 (2)	0.006 (2)	−0.002 (3)
C2	0.054 (3)	0.056 (4)	0.088 (5)	0.003 (2)	0.001 (3)	−0.021 (4)
C3	0.059 (3)	0.048 (3)	0.110 (6)	−0.003 (2)	−0.006 (3)	0.005 (4)
C4	0.051 (3)	0.083 (4)	0.081 (5)	0.001 (3)	0.006 (3)	0.025 (5)
C5	0.050 (3)	0.075 (4)	0.055 (4)	0.008 (3)	0.008 (3)	0.005 (3)
C6	0.034 (2)	0.069 (3)	0.046 (3)	0.002 (2)	0.003 (2)	−0.002 (3)
C7	0.035 (2)	0.058 (3)	0.033 (3)	0.004 (2)	0.0013 (19)	−0.009 (3)
C8	0.043 (2)	0.056 (3)	0.038 (3)	0.003 (2)	−0.004 (2)	−0.005 (3)
C9	0.042 (2)	0.045 (2)	0.029 (2)	0.0030 (18)	0.0034 (19)	−0.007 (2)
C10	0.047 (2)	0.062 (3)	0.036 (3)	0.001 (2)	0.004 (2)	−0.009 (3)
C11	0.041 (2)	0.042 (3)	0.047 (3)	0.0072 (19)	0.001 (2)	−0.015 (2)
C12	0.054 (3)	0.055 (3)	0.057 (4)	0.010 (2)	−0.014 (2)	−0.004 (3)
C13	0.050 (3)	0.056 (3)	0.097 (5)	−0.001 (2)	−0.019 (3)	−0.012 (4)
C14	0.055 (3)	0.062 (4)	0.106 (6)	−0.001 (2)	0.003 (3)	−0.001 (4)
C15	0.065 (3)	0.064 (3)	0.057 (4)	0.008 (3)	0.005 (3)	−0.003 (3)
C16	0.051 (2)	0.044 (3)	0.047 (3)	0.009 (2)	0.001 (2)	−0.010 (3)
C17	0.087 (4)	0.068 (4)	0.045 (4)	−0.005 (3)	−0.015 (3)	0.005 (3)
C18	0.044 (2)	0.049 (3)	0.030 (2)	0.0022 (19)	0.007 (2)	−0.004 (2)
N1	0.052 (2)	0.071 (3)	0.043 (3)	0.003 (2)	0.0102 (18)	−0.008 (2)
N2	0.041 (2)	0.072 (3)	0.057 (3)	0.0055 (18)	0.001 (2)	−0.007 (3)
O1	0.068 (2)	0.053 (2)	0.045 (2)	0.0031 (17)	0.0091 (18)	−0.0161 (19)
O2	0.070 (2)	0.062 (2)	0.042 (2)	0.0082 (16)	0.0097 (17)	−0.012 (2)

### Geometric parameters (Å, º)

**Table:**

C1—C2	1.379 (7)	C10—O1	1.405 (6)
C1—O1	1.381 (7)	C10—H10A	0.9700
C1—C6	1.404 (7)	C10—H10B	0.9700
C2—C3	1.370 (10)	C11—C16	1.388 (7)
C2—H2	0.9300	C11—C12	1.403 (6)
C3—C4	1.399 (9)	C12—C13	1.376 (7)
C3—H3	0.9300	C12—H12	0.9300
C4—C5	1.347 (8)	C13—C14	1.383 (10)
C4—H4	0.9300	C13—H13	0.9300
C5—C6	1.406 (7)	C14—C15	1.365 (8)
C5—H5	0.9300	C14—H14	0.9300
C6—C7	1.433 (7)	C15—C16	1.381 (7)
C7—N1	1.271 (6)	C15—H15	0.9300
C7—C9	1.523 (6)	C16—C17	1.495 (7)
C8—O2	1.461 (6)	C17—H17A	0.9600
C8—C11	1.497 (6)	C17—H17B	0.9600
C8—C9	1.537 (6)	C17—H17C	0.9600
C8—H8	0.9800	C18—N2	1.131 (5)
C9—C18	1.487 (5)	N1—O2	1.410 (5)
C9—C10	1.512 (7)		
			
C2—C1—O1	118.0 (5)	O1—C10—H10A	109.3
C2—C1—C6	120.6 (6)	C9—C10—H10A	109.3
O1—C1—C6	121.5 (4)	O1—C10—H10B	109.3
C3—C2—C1	119.8 (6)	C9—C10—H10B	109.3
C3—C2—H2	120.1	H10A—C10—H10B	108.0
C1—C2—H2	120.1	C16—C11—C12	119.8 (4)
C2—C3—C4	120.4 (5)	C16—C11—C8	121.2 (4)
C2—C3—H3	119.8	C12—C11—C8	118.9 (5)
C4—C3—H3	119.8	C13—C12—C11	120.5 (6)
C5—C4—C3	120.1 (6)	C13—C12—H12	119.8
C5—C4—H4	119.9	C11—C12—H12	119.8
C3—C4—H4	119.9	C12—C13—C14	119.5 (5)
C4—C5—C6	121.0 (6)	C12—C13—H13	120.3
C4—C5—H5	119.5	C14—C13—H13	120.3
C6—C5—H5	119.5	C15—C14—C13	119.6 (6)
C1—C6—C5	118.0 (5)	C15—C14—H14	120.2
C1—C6—C7	118.2 (5)	C13—C14—H14	120.2
C5—C6—C7	123.7 (5)	C14—C15—C16	122.5 (6)
N1—C7—C6	127.5 (4)	C14—C15—H15	118.7
N1—C7—C9	113.8 (4)	C16—C15—H15	118.7
C6—C7—C9	118.4 (4)	C15—C16—C11	118.0 (5)
O2—C8—C11	110.1 (4)	C15—C16—C17	119.9 (5)
O2—C8—C9	103.1 (4)	C11—C16—C17	122.1 (4)
C11—C8—C9	117.8 (4)	C16—C17—H17A	109.5
O2—C8—H8	108.5	C16—C17—H17B	109.5
C11—C8—H8	108.5	H17A—C17—H17B	109.5
C9—C8—H8	108.5	C16—C17—H17C	109.5
C18—C9—C10	109.7 (3)	H17A—C17—H17C	109.5
C18—C9—C7	108.9 (4)	H17B—C17—H17C	109.5
C10—C9—C7	107.6 (3)	N2—C18—C9	178.8 (5)
C18—C9—C8	113.6 (3)	C7—N1—O2	109.1 (4)
C10—C9—C8	117.3 (4)	C1—O1—C10	118.3 (4)
C7—C9—C8	98.7 (4)	N1—O2—C8	107.9 (3)
O1—C10—C9	111.5 (4)		
			
O1—C1—C2—C3	179.9 (4)	C18—C9—C10—O1	62.9 (5)
C6—C1—C2—C3	0.4 (7)	C7—C9—C10—O1	−55.5 (4)
C1—C2—C3—C4	−0.8 (8)	C8—C9—C10—O1	−165.5 (4)
C2—C3—C4—C5	0.9 (8)	O2—C8—C11—C16	−140.8 (4)
C3—C4—C5—C6	−0.7 (7)	C9—C8—C11—C16	101.4 (5)
C2—C1—C6—C5	−0.2 (6)	O2—C8—C11—C12	42.6 (5)
O1—C1—C6—C5	−179.7 (4)	C9—C8—C11—C12	−75.2 (6)
C2—C1—C6—C7	177.9 (4)	C16—C11—C12—C13	−1.1 (7)
O1—C1—C6—C7	−1.5 (6)	C8—C11—C12—C13	175.5 (5)
C4—C5—C6—C1	0.3 (7)	C11—C12—C13—C14	−1.5 (8)
C4—C5—C6—C7	−177.7 (4)	C12—C13—C14—C15	2.6 (8)
C1—C6—C7—N1	163.8 (4)	C13—C14—C15—C16	−1.0 (9)
C5—C6—C7—N1	−18.2 (7)	C14—C15—C16—C11	−1.5 (7)
C1—C6—C7—C9	−10.4 (5)	C14—C15—C16—C17	179.5 (5)
C5—C6—C7—C9	167.6 (4)	C12—C11—C16—C15	2.6 (6)
N1—C7—C9—C18	103.9 (4)	C8—C11—C16—C15	−174.0 (4)
C6—C7—C9—C18	−81.1 (4)	C12—C11—C16—C17	−178.5 (5)
N1—C7—C9—C10	−137.2 (4)	C8—C11—C16—C17	5.0 (6)
C6—C7—C9—C10	37.8 (5)	C6—C7—N1—O2	−175.9 (4)
N1—C7—C9—C8	−14.8 (5)	C9—C7—N1—O2	−1.4 (5)
C6—C7—C9—C8	160.2 (4)	C2—C1—O1—C10	162.0 (4)
O2—C8—C9—C18	−91.3 (4)	C6—C1—O1—C10	−18.6 (6)
C11—C8—C9—C18	30.2 (7)	C9—C10—O1—C1	48.4 (5)
O2—C8—C9—C10	138.9 (4)	C7—N1—O2—C8	18.6 (4)
C11—C8—C9—C10	−99.6 (5)	C11—C8—O2—N1	−153.6 (3)
O2—C8—C9—C7	23.9 (4)	C9—C8—O2—N1	−27.1 (4)
C11—C8—C9—C7	145.4 (4)		
